# Human rhinovirus C: a newly discovered human rhinovirus species

**DOI:** 10.3134/ehtj.10.002

**Published:** 2010-10-04

**Authors:** S K P Lau, C C Y Yip, P C Y Woo, K-Y Yuen

**Affiliations:** 1 State Key Laboratory of Emerging Infectious Diseases, The University of Hong Kong, Hong Kong, China; 2 Research Centre of Infection and Immunology, The University of Hong Kong, Hong Kong, China; 3 Carol Yu Center for Infection, The University of Hong Kong, Hong Kong, China; 4 Department of Microbiology, The University of Hong Kong, Hong Kong, China

## Abstract

Although often ignored, human rhinoviruses (HRVs) are the most frequent causes of respiratory tract infections (RTIs). A group of closely related novel rhinoviruses have recently been discovered. Based on their unique phylogenetic position and distinct genomic features, they are classified as a separate species, HRV-C. After their discovery, HRV-C viruses have been detected in patients worldwide, with a reported prevalence of 1.4–30.9% among tested specimens. This suggests that the species contribute to a significant proportion of RTIs that were unrecognized in the past. HRV-C is also the predominant HRV species, often with a higher detection rate than that of the two previously known species, HRV-A and HRV-B. HRV-C infections appear to peak in fall or winter in most temperate or subtropical countries, but may predominate in the rainy season in the tropics. In children, HRV-C is often associated with upper RTIs, with asthma exacerbation and wheezing episodes being common complications. The virus has also been detected in children with bronchitis, bronchiolitis, pneumonia, otitis media, sinusitis and systemic infections complicated by pericarditis. As for adults, HRV-C has been associated with more severe disease such as pneumonia and exacerbation of chronic obstructive pulmonary disease. However, larger clinical studies with asymptomatic controls are required to better define the significance of HRV-C infection in the adult population. On the basis of VP4 sequence analysis, a potential distinct subgroup within HRV-C has also been identified, although more complete genome sequences are needed to better define the genetic diversity of HRV-C.

## Introduction

Human rhinoviruses (HRVs) are the most frequent causes of acute respiratory tract infections (RTIs). They are small, nonenveloped, single-stranded, positive-sense, RNA viruses that are now classified within the genus *Enterovirus* of the eight genera belonging to the family Picornaviridae. Traditionally, they have been associated only with mild upper respiratory tract infections (URTIs). Most ‘classical’ HRVs have a relatively low optimal temperature for growth (33°c), reflecting their adaptation to the human nasopharynx and association with URTIs. However, recent studies have shown that they are increasingly associated with more severe diseases such as pneumonia—especially in infants, the elderly and immunocompromised patients.^1^–^3^ ‘Classical’ HRVs consist of more than 100 distinct serotypes, which appear to correlate with VP1 gene sequences.^4^ The different serotypes can be further classified according to receptor specificity, antiviral susceptibility and nucleotide sequence identities.^4^ On the basis of VP4/VP2 gene sequence analysis, all serotypes except HRV87 were classified into two species, HRV-A with 74 serotypes, and HRV-B with 25 serotypes.^5^–^8^

Because the etiological agents of a significant proportion of RTIs remain unknown,^9^, ^10^ intensive research efforts were carried out in the past few years to identify novel respiratory pathogens that could be responsible. Apart from the recent discovery of human metapneumovirus,^11^ severe acute respiratory syndrome coronavirus,^12^ human coronavirus NL63 (HCoV-NL63),^13^, ^14^ human coronavirus HKU1 (HCoV-HKU1)^15^–^18^ and human bocavirus (HBoV),^18^–^20^ several research groups have previously reported the detection of novel HRV genotypes in respiratory tract specimens from patients in the United States, Australia and China.^21^–^25^ Analysis of the complete genome sequences suggested that these newly identified HRV genotypes belonged to a potentially novel HRV species, human rhinovirus C (HRV-C), with genome features distinct from HRV-A and HRV-B.^24^, ^26^ HRVs closely related to HRV-C have subsequently been found in patients from various countries, suggesting that these viruses are circulating worldwide and represent an important cause of respiratory disease.^25^, ^27^–^41^ In this review, we summarize current knowledge of the epidemiology, clinical features, genome features, molecular diagnosis and genetic diversity of this novel HRV species.

## Methods

Keywords including ‘rhinovirus’, ‘new’, ‘novel’, ‘HRV’ and ‘HRV-C’ were used in MEDLINE searches. The search results were then manually screened to include literature on the newly described HRV-C species or related strains.

### Human rhinovirus C: discovery of a novel HRV species

In 2006, new rhinovirus genotypes were identified in respiratory samples collected from patients in Queensland and New York City.^21^, ^22^ Because only partial VP4/VP2 sequences were available, the phylogenetic positions of these new genotypes could not be ascertained then, and the strains found in New York were designated HRV-NY. After complete polyprotein gene sequencing a Queensland strain, designated HRV-QPM, was classified as a subgroup of HRV-A, HRV-A2.^23^ In a previous study of HBoV infections in children admitted to hospital,^24^ we also identified the presence of HRV sequences that did not cluster with either HRV-A or HRV-B species. Upon VP4 sequence analysis, these HRV strains from New York, Queensland and Hong Kong fell into a distinct cluster away from HRV-A and HRV-B, suggesting that they represent a novel clade of HRVs. To better ascertain their phylogenetic position and genome structure, we carried out complete genome sequence analysis on three of strains found by us, which revealed distinct genome features supporting the classification of HRV-C as a separate species.^24^ As a result of limited sequence data available in earlier studies, it was uncertain whether HRV-Cs represent only novel genotypes or a sublineage within HRV-A, so these virus strains were given different names (Table 1). However, based on the currently available sequence data, HRV-C is now proposed by the International Committee on Taxonomy of Viruses (http://talk.ictvonline.org/media/p/ 1201.aspx) as a new HRV species in the genus *Enterovirus,* family *Picornaviridae*, order *Picornavirales*.

**Table 1:** Published reports on detection of HRV-C from clinical samples

*References*	*Country/region*	*Age range of study population (median)*	*Clinical syndromes*	*Method of detection*	*Target(s) for species classification*	*Type of sample*	*No. of tested samples*	*No. (%) of samples positive for HRV-C*	*Designated virus strain/clade name*
Arden *et al*.^21^	Australia	1 day to 80.3 years (1.2 years)	Acute RTI	RT–PCR	VP4/VP2	92.4% NPA	315	9 (2.9)	HRV-A2
Lamson *et al.* 22	USA	4 months to 98 years (25 years)	Influenza-like illness	MassTag PCR	VP4	Oropharyngeal, NP, nasal swabs	151	8 (5.3)	HRV-NY
McErlean *et al.* 23	Australia	1 day to 80 years (1.3 years)	Acute RTI	Real-time RT-PCR	VP4, VP2, VP1	92% NPA	1244	17(1.4)	HRV-QPM
Lau *et al.* 24	Hong Kong	<18 years	Acute RTI	RT-PCR	VP4	NPA	203	21 (10.3)	HRV-C
Kistler *et al.* 25	USA	Adults	RTI±asthma	Virochip	VP4/VP2	Nasal lavage	82	5 (6.1)	HRV 'X'
Renwick *et al.* 27	Cermany	14 days to 5 years (10 months)	Acute RTI	Microarray MassTag PCR	VP4/VP2	NPA	97	30 (30.9)	HRV X
Lee *et al.* 28	USA	=1 year	Frequent respiratory illnesses	Respiratory Multicode Assay	5′-NCR	Nasal lavage	181	9 (5.0)	HRVC
Briese *et al.* 29	South África, Côte d'lvoire, Nepal, India, Australia, Denmark, Spain	0.4 months to 56 years	Acute RTI	MassTag PCR	VP4/VP2	Respiratory specimens	326	23 (7.1)	Novel genotype
Dominguez *et al*.^30^	USA	Mean 40.1 ±33.4 months	Respiratory symptoms	MassTag PCR	VP4/VP2	NP washes	44	9 (20.5)	HRV-CO
Savolainen-Kopra *et al*.^31^	Finland	<2 years	Acute otitis media	RT–PCR	VP4/VP2	NPA, middle ear fluid	92 in 38 AOM events	1 6 (1 7.4)	HRV-C PNC
Kiang *et al.* 32	USA	Pediatric and adult patients	Acute RTI	RT–PCR	5′-NCR	NPS, NPA, ETA, BAL, Pleural fluid	24 positive for HRV	5 (20.8)	Genogroup C
Xiang *et* a*l*.^33^	China	1 month to 15 years (10 months)	LRTI	RT–PCR	VP4/VP2	NPA	258	14 (5.4)	HRV-C BCH
Miller *et al.* 34	USA	<5 years	Acute RTI or fever	RT–PCR	VP4/VP2	Nasal/throat swab	1052	77 (7.3)	HRVC
Khetsuriani *et al.* 35	USA	=2 years	Asthma	Seminested RT-PCR	VP1	NPS	142 (65 cases+77 controls)	8 (5.6)	Genogroup C
Tapparel *et al.* 36 (case report)	Switzerland	14 months	LRTI with pericarditis	Real-time RT-PCR	VP1	Plasma, BAL, pericardial effusion, stool	NA	NA	HRV-C
Han *et al.* 37	South Korea	1 month to 158 months (14 months)	LRTI	RT–PCR	5′-NCR	NPA	470	17(3.6)	HRV-C KR
Piralla *et al.* 38	Italy	Pediatric and adult patients	Acute RTI	Real-time RT-PCR	5′-NCR, VP4/VP2	NPA	301	28 (9.3)	HRV-C PV
Linsuwanon *et al.* 39	Thailand	Pediatric patients	Acute RTI	RT–PCR	VP4/2	NPA	289	50 (17.3)	HRV-C
Lau *et al.* 40	Hong Kong	Pediatric and adult patients	Acute RTI	RT–PCR	VP4	NPA	1200	91 (7.6)	HRV-C
Huang *et* a*l*.^41^	Shanghai	Pediatric patients	LRTI	RT–PCR	5′-NCR, VP4/VP2	NPS	827	34 (4.1)	HRV-C

Abbreviations: HRV-C, human rhinovirus C; RTI, respiratory tract infections; LRTI, lower respiratory tract infections; NA, not applicable; NPA, nasopharyngeal aspirate; NPS, nasopharyngeal swab, ETA, endotracheal aspirate; BAL, bronchoalveolar lavage; RT–PCR, reverse transcription–PCR.

### Epidemiology of HRV-C

Within three years since its first descriptions up to July 2009, 20 reports have been published describing the detection of HRV-C in clinical samples of patients from countries in Africa, Asia, Australia, Europe and America.^21^–^25^, ^27^–^41^ Most reports were based on respiratory samples taken from patients with respiratory illness, with the reported prevalence of HRV-C ranging from 1.4 to 30.9% among tested specimens. The difference in reported prevalence among available studies is likely due to differences among patient groups, specimen types and detection methods. Nevertheless, in most studies, HRV-C was detected in >5% of tested specimens, suggesting an important role for the virus in RTIs worldwide. Apart from sporadic infections, HRV-C probably causes frequent outbreaks of respiratory illness in the community and institutions. In our recent study, 12% of cases of HRV-C infections were acquired during institutionalization or admission to hospital.^40^ In incidents of community-acquired illness, clusters of cases were linked to the same strains of HRV-C within a short period of time and there were frequent reports of contact among patients who suffered from a similar illness.^40^

HRV-C appears to dominate among HRVs implicated in human RTIs. Among the 26 nasopharyngeal aspirates (NPAs) tested positive for HRV in our earlier study, 21 (81%) belonged to HRV-C whereas five (19%) belonged to HRV-A; HRV-B was not detected.^24^ In a recent study that included 220 NPAs positive for HRV, HRV-A was the most prevalent species (50%), whereas HRV-C accounted for 41% and HRV-B accounted for 8% of respiratory samples, suggesting that HRV-A and HRV-C are more prevalent than HRV-B in Hong Kong.^40^ In a number of studies in other countries, HRV-C was found to be the predominant HRV species as it was identified in 50–73% of tested samples, whereas HRV-B was the least common species.^21^, ^22^, ^27^, ^39^ However, the sensitivity of the various assays used to spot different HRV species was uncertain in many of the studies. In a study from the United States, HRV-C was suspected as the cause of almost half of all HRV-associated hospitalizations.^34^ On the basis of these data, it can be concluded that this newly described HRV species likely contributes to a significant proportion of HRV infections that were unrecognized in the past.

Human rhinoviruses are known as a cause of epidemics in early fall and late spring. In some studies, which include clinical specimens collected throughout the year, HRV-C appears to show seasonal patterns of infection. In Hong Kong, a subtropical city, infections caused by HRVs, including HRV-C, occur throughout the year, although a higher incidence has been observed during the fall and winter months.^24^, ^40^ In a study from Beijing, HRV-C was also detected in specimens collected in the fall (October–December) but not during summer (July–September).^33^ A recent study from Thailand has shown that HRV-C can be found throughout the year but predominates in the rainy season,^39^ whereas in South Korea it has been detected mostly in the spring.^37^ As for Australia, HRV-C appeared to reach peak prevalence in the winter, but was also detected in spring and summer.^23^ In a study from the United States, HRV-C prevalence peaked in October (early fall).^34^ Therefore, it appears that HRV-C shows seasonality, with peaks in fall or winter in most temperate or subtropical countries, but a possible peak incidence during the rainy season in the tropics. As peaks in fall and winter have also been observed in HRV-A and HRV-B infections, infections with these rhinoviruses may show similar seasonality.^40^ We recently reported an apparent alternating disease activity between HRV-A and HRV-C, although studies of longer duration are required to confirm this phenomenon, which may be due to viral interference or cross-serological protection similar to that seen with human parainfluenza viruses.^40^, ^42^

Despite the high prevalence of HRV-C and other HRVs detected in various studies, it is important to note that few data were available on the incidence of co-infections. In our earliest study, HRV-C strains were identified during a search for co-pathogens in HBoV infections.^24^ In a recent study that involved patients from Thailand, the frequency of coinfection with HRV-C and other respiratory viral pathogens—including HBoV, adenovirus, polyomavirus, influenza virus, parainfluenza virus, respiratory syncytial virus and human metapneumovirus—was as high as 40%.^39^ In a recent report from Shanghai, co-infection by HRV-A and HRV-B or HRV-C was also observed.^41^ In fact, co-detection of multiple viruses in respiratory infections has been increasingly frequent as a result of the availability of more accurate detection assays, and the development of microarrays and multiplex detection assays.^25^, ^43^–^48^ The concept of virusassociated RTIs is likely to be revolutionized in the near future, and the role of respiratory viruses will probably be redefined.

### Clinical features of HRV-C infections

HRV-C is now known to be associated with both mild URTIs and more severe lower respiratory tract infections (LRTIs) in children and adults. In the earliest studies, HRV-C was detected in patients with influenza-like illness, infants with bronchiolitis and children admitted to hospital for acute respiratory illness.^21^–^24^ Although children with HRV-C infections often presented with URTIs, complications were not uncommon and included asthmatic exacerbations or febrile wheeze triggered by the virus infection.^23^, ^24^, ^34^, ^35^, ^39^, ^40^ A study from the United States found that children with HRV-Cs were more likely than those with HRV-As to have a diagnosis of asthma on discharge from hospital.^34^ In a recent study by us, it was found that wheezing episodes were also more common in patients infected with HRV-C and HRV-A than those who had HRV-B infections.^40^ HRV-C has been detected frequently in children with LRTIs including bronchitis, bronchiolitis and pneumonia.^23^, ^24^, ^27^, ^33^, ^37^, ^39^, ^40^ This suggests that these viruses are also associated with more severe respiratory disease. However, a recent report did not find that HRV-C strains had greater clinical impact than HRV-A or HRV-B on respiratory compromise in children with LRTI.^41^ HRV-C has also been detected in children with acute otitis media and acute sinusitis, in addition to RTIs.^24^, ^40^ This fact is supported in a recent study in Finland, which reported the presence of the virus in the middle-ear fluid of young children with acute otitis media.^31^ In a recent case report from Switzerland, HRV-C was detected in the bronchoalveolar lavage specimen, pericardial fluid, plasma and stool of a 14-month-old boy with an LRTI complicated by severe pericarditis.^36^ These reports suggest that the new HRV species can cause extrarespiratory and systemic infection, with serious complications such as pericarditis.

The significance of HRV-C infection in the adult population is less well studied. This is probably due to the lower hospitalization and sampling rate for respiratory illness in adults, and the lack of clinical data even in studies where samples from adults are included.^21^, ^22^, ^29^, ^32^ In reports where a brief clinical diagnosis was provided, HRV-C was detected in adults with exacerbation of chronic obstructive pulmonary disease,^23^ asthma,^25^ URTIs and LRTIs.^38^ In a recent study by us that aimed to better define the role of HRV-C in adult respiratory illness, eight (62%) of the 13 adults with HRV-C infections had pneumonia, a rate significantly higher than that observed with HRV-A infections in the study population.^40^ The other four patients presented with URTI and one with exacerbation of chronic obstructive pulmonary disease.^40^ Further clinical studies that involve more adult patients and asymptomatic controls are required to examine whether HRV-C could be more virulent than other HRVs.

### Distinct genome features of HRV-C

Genomic studies on HRVs have been limited until recently, with only five HRV-A and one HRV-B genomes published before 2007. Although 97 and 31 complete genome sequences are now available for HRV-A and HRV-B, respectively,^49^–^58^ only seven genomes or complete polyprotein coding regions of HRV-C have been sequenced up to the time of writing this article: three from Hong Kong strains (HRV-C 024, 025 and 026), two from California (NAT001 and NAT045), one from New York (NY1078) and one from Australia (HRV-QPM).^23^–^25^, ^49^ The genome organization of HRV-C is typical of *Picornaviridae*, comprising a 5′-noncoding region (5′-NCR), a single open reading frame encoding a single polyprotein and a 3′-NCR before a polyadenylated tract. However, the genome of HRV-C was the shortest among other reported HRVs and HEVs, a result of several deletions.^23^, ^24^ Although the P1 region of HRV-C possessed higher amino-acid identities to that of HRV-A than to the P1 region of HRV-B, the P3 region possessed higher amino-acid identities to that of HRV-B than to the P3 region of HRV-A. The predicted amino-acid sequence of the HRV-C polyprotein shared only 51–52% and 47–48% with that of HRV-A and HRV-B, respectively.^23^, ^24^ Phylogenetic analysis of the 5′-NCR region and the predicted proteins revealed that HRV-C strains formed a distinct cluster away from HRV-A and HRV-B, which indicates that they represent a novel HRV species. Although HRV-C is a newly described HRV species, molecular dating analysis using sequences of the VP4/2 coding regions indicated that these viruses have been circulating for at least 250 years, with an estimated evolutionary rate of 6.6×10^−4^ substitutions per site per year.^29^

HRV-C shows several distinct genomic features that further support its classification as a separate species. First, a unique putative cleavage site was identified between VP4 and VP2.^24^ Second, major insertions and deletions were observed in VP1, which is shorter than that of other HRVs, especially in regions that were important for neutralization in HRV-A and HRV-B.^23^, ^24^ Third, the conserved amino-acid residues within VP1 that determine receptor binding of HRV-A and HRV-B were found to have frequent substitutions in HRV-C.^24^ Using structural homology modeling, it was also found the VP1 of HRV-C possesses structural disparities within sites for receptor binding of HRV-A and HRV-B.^26^ These suggest that HRV-C may use a different cellular receptor, reflected in its failure to grow in traditional cell cultures. In addition, a novel distinct *cis*-acting replication element located in the VP2 region has been identified in HRV-C, supporting its classification as a separate species.^59^ By completing the genome sequences for all known HRV serotypes, a recent study also confirmed species-specific sequence and RNA structure elements that differentiate HRV-C from HRV-A and HRV-B.^49^ Although HRV-A strains are currently susceptible to the antiviral drug pleconaril, analysis of the genome sequence data suggested that HRV-C may be resistant to this drug, in view of the presence of Phe_152_ and Thr_191_ in the VP1 of some reported strains.^23^, ^24^ However, this would need to be verified by *in vitro* assays when cell culture systems are established. The use of reverse genetic approaches may overcome the difficulties in isolating HRV-C.

### Molecular detection and genetic diversity of HRV-C

5′-NCR and VP4/VP2 regions were the most commonly used targets for detection of HRV-C, although the 5′-NCR region appears to be more sensitive for HRV detection.^32^, ^37^ In one study, VP4/VP2 RT-PCR failed to detect all 24 samples with HRVs using cDNA templates identical to those used for 5′-NCR RT-PCR.^32^ However, 5′-NCR sequence is not suitable for assignment of phylogenetic relationships.^22^, ^60^ To better determine the prevalence of HRVs in respiratory specimens, it is important that 5′-NCR regions should be used for detection and VP4/VP2 or VP1 sequences for more accurate species identification. Because VP4 is a highly conserved protein, the use of amino-acid sequences may underestimate the diversity and number of HRV strains as compared with nucleotides sequences. However, it remains to be determined which should be the valid approach for representation of different HRV-C strains.

In addition to the commonly used PCR methodology, other molecular detection methods have also been used to detect HRV-C viruses. In a study from the United States, Virochip was used for virus detection.^25^ Virochip is a DNA microarray that contains the most conserved sequences of all known viruses identified in humans, animals, plants and microbes.^46^ This method can detect new members of known virus families by cross-hybridization, which offers a significant advantage compared to PCR-based methods.^46^, ^47^ In another study, also from the United States, respiratory multicode assay (RMA) was used to detect viruses.^28^ RMA is a high-throughput, multiplex PCR-microsphere flow cytometry assay designed for comprehensive detection of common respiratory viruses and consists of five main steps, all of which take place in the same microwell.^48^ Apart from having the capacity to detect new viruses, these assays also allow simultaneous detection of multiple viruses from the same sample, which may provide better information on rates of co-infection.

Real-time RT-PCR has been used to measure the viral load of HRV-C in clinical specimens. In a study from Italy, the median peak viral load of 28 patients with HRV-C infection was 4.5×10^6^ RNA copies per ml in NPA, which is slightly higher than that seen in 45 patients with HRV-A infection (2.2×10^6^ RNA copies per ml) and in 12 patients with HRV-B infection (1.6×10^5^ RNA copies per ml); however, the difference was not statistically significant.^38^ In a case report from Switzerland about HRV-C LRTI complicated by pericarditis, the viral load was significantly higher in the bronchoalveolar lavage specimen compared with that in plasma and stool specimens, consistent with the virus’ association with RTIs.^36^

**Figure 1:**
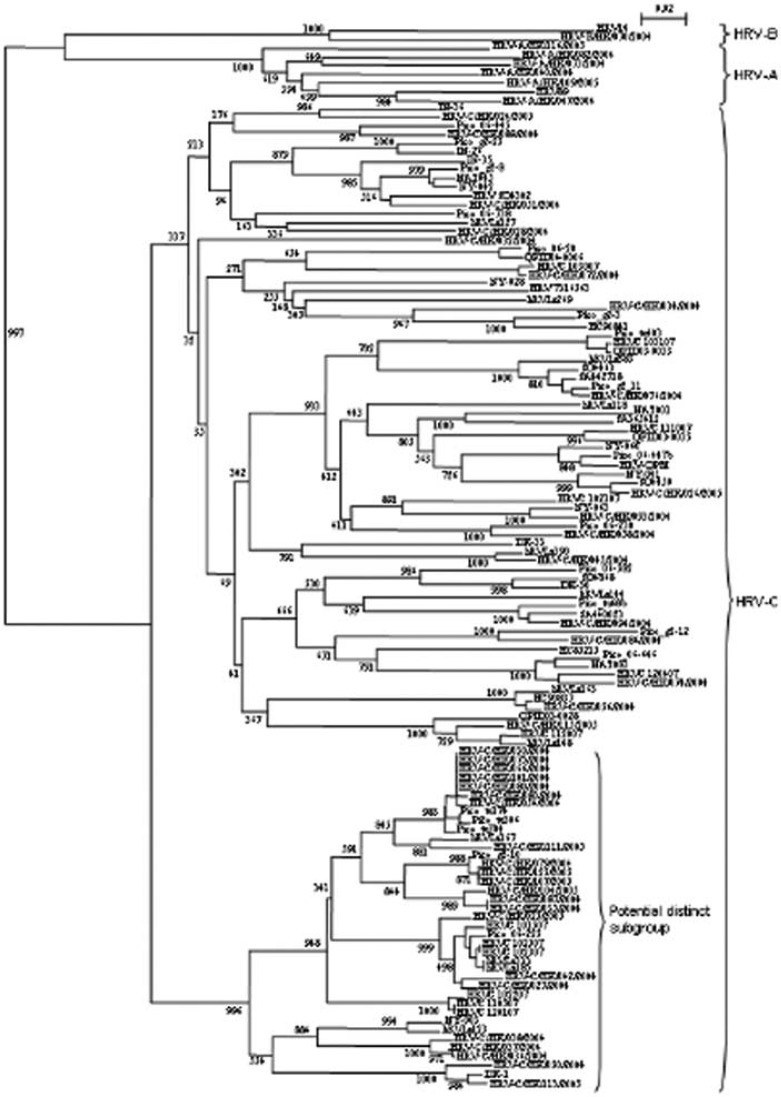
Phylogenetic tree of the VP4 region of HRV-C strains detected in different countries, showing the presence of a potential distinct subgroup. 201 nucleotide positions in each VP4 region were included in the analysis. The tree was constructed by a neighbor-joining method and bootstrap values were calculated from 1000 trees. The scale bar indicates the estimated number of substitutions per 50 nucleotides. Strains from Hong Kong are indicated by HRV-C/HK/strain no./year of detection. The GenBank accession numbers of the previously published sequences are as follows: HRV-QPM, EF186077; HRV89, A10937; HRV14, NC_001490; DK-1, EU697851; DK-30, EU697837; DK-33, EU697833; HC85215, EU697846; HC90837, EU697856; HC90841, EU697863; hRVLz118, EU822834; hRVLz123, EU822836; hRVLz127, EU822838; hRVLz144, EU822840; hRVLz148, EU822841; hRVLz163, EU822842; hRVLz167, EU822843; hRVLz185, EU822847; hRVLz269, EU822856; hRVLz333, EU822869; hRVLz383, EU822878; hRVLz390, EU822879; IN-26, EU697865; IN-35, EU697845; IN-36, EU697839; HRV 7316563, EU697850; NY-003, DQ875929; NY-028, DQ875931; NY-041, DQ875921; NY-042, DQ875926; NY-060, DQ875928; NY-063, DQ975924; SA365412, EU697852; SA440023, EU697829; SA442718, EU697828; SO4302, EU697869; SO4450, EU697835; SO4463, EU697854; SO4868, EU697854; NAT001, EF077252; NAT045, EF077253; NAT083, EF077264; QPID03-0035, EU155152; QPID03-0033, EU155153; QPID03-0028, EU155154; QPID04-0006, EU155158; HRVC 102307, EU687518; HRVC 102507, EU687523; HRVC 101507, EU687515; HRVC 120107, EU687527; HRVC102207, EU687516; HRVC 110507, EU687522; HRVC 103007, EU687519; HRVC 112007, EU687525; HRVC 103107, EU687520; HRVC 102107, EU687517; HRVC 111007, EU687524; HRVC 120407, EU687528; Pico tu304, EU081791; Pico tu306, EU081793; Pico tu403, EU081795; Pico 06-445, EU081796; Pico tu68b, EU081797; Pico g2-10, EU081798; Pico g2-11, EU081799; Pico 06-20, EU081800; Pico g2-12, EU081801; Pico g2-25, EU081802; Pico 06-447b, EU081806; Pico 06-230, EU081807; Pico 06-582, EU081809; Pico g2-3, EU081810; Pico 06-646, EU081811; Pico g2-8, EU081812; Pico 06-738, EU081813; Pico tu174, EU081814; Pico 06-225, EU081815.

Currently, data on serotype recurrence of HRVs and their seasonal distribution are very limited. In our recent report, recurrent infections by different HRV strains of the same patient were observed within a short period of time.^40^ Whereas numerous HRV strains belonging to the HRV-C species have been identified, a huge genetic diversity was detected among strains within the species, and this may help the virus evade immune protection. In our study, the VP4 sequences of the identified HRV-C strains showed 67.2–82.1% nucleotide identities to that of our reference HRV-C strain 024.^40^ By phylogenetic analysis of available VP4 sequences of HRV-C, we also identified a potential distinct subgroup of strains within HRV-C Figure 1.^40^ Interestingly, HRV-C strains presenting divergent 5′-NCR sequences were also observed in the past,^28^, ^61^ and a recent report proposed to subdivide HRV-C in two distinct subspecies based on 5′-NCR regions.^41^ However, as only 5′-NCR regions were studied for some of the previously reported strains, it remains to be determined if these subspecies correspond to the subgroup we identified based on VP4 sequences. More complete genome sequences of strains from these potential subgroups of HRV-C and successful isolation of different HRV-C genotypes would be required to ascertain the existence of subspecies, and to study their genetic diversity and cross-serological reactivity.

## Concluding remarks

Because HRVs were historically considered to have little health impact and clinical significance, the possible existence of novel species and the relative importance and classification of the different species have been poorly investigated.^62^ Recent studies have shown that in fact HRVs are more important than respiratory syncytial virus as a cause of respiratory virus-associated hospital admissions, and as predictors of recurrent wheezing in children in large cohorts.^2^, ^63^ The discovery of HRV-C as a novel species with diverse genotypes, and its prevalence in human respiratory samples, has ushered a new era in HRV research, by uncovering viruses that account for a significant proportion of previously undiagnosed respiratory illness. However, our understanding of this novel HRV species has been hindered to date by a failure to culture these viruses in cell lines. Therefore, further studies on HRV-C are required to better understand the viruses’ genetic diversity, mechanism of immune evasion, virulence and antiviral susceptibilities. We suggest that these studies should focus on analysis of more HRV-C genome sequences, pathogenesis and the development of culture systems.
